# 3,4-Dimethyl­anilinium 4-methyl­benzene­sulfonate

**DOI:** 10.1107/S160053681103892X

**Published:** 2011-09-30

**Authors:** Shi Juan Wang

**Affiliations:** a Department of Applied Chemistry, Nanjing College of Chemical Technology, Nanjing 210048, People’s Republic of China

## Abstract

In the crystal structure of the title compound, C_8_H_12_N^+^·C_7_H_7_O_3_S^−^, N—H⋯O hydrogen bonds link the cations and anions into ribbons parallel to the *c* axis. N—H⋯S inter­actions also occur.

## Related literature

For background to protonated amines, see: Tong & Whitesell (1998 ▶); Shanker (1994 ▶). For closely related structures, see: Hemissi *et al.* (2001 ▶); Bouacida (2008 ▶); Singh *et al.* (2002 ▶).
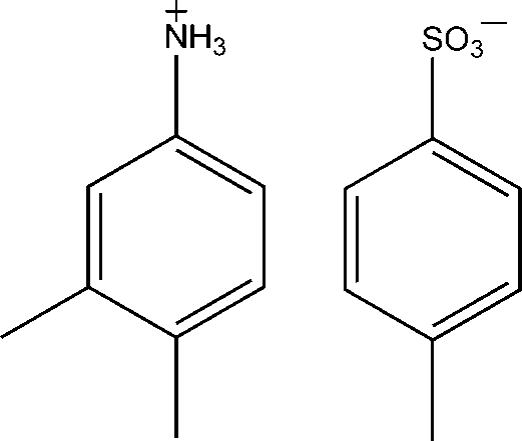


## Experimental

### 

#### Crystal data

C_8_H_12_N^+^C_7_H_7_O_3_S^−^
*M* *_r_* = 293.37
Monoclinic, 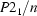

*a* = 12.373 (3) Å
*b* = 7.3011 (15) Å
*c* = 17.556 (4) Å
β = 106.88 (3)°
*V* = 1517.7 (5) Å^3^
*Z* = 4
Mo *K*α radiation
μ = 0.22 mm^−1^
*T* = 293 K
0.20 × 0.20 × 0.20 mm

#### Data collection

Rigaku Mercury2 diffractometer
Absorption correction: multi-scan (*CrystalClear*; Rigaku, 2005 ▶) *T*_min_ = 0.825, *T*_max_ = 1.000
14838 measured reflections
3434 independent reflections
2608 reflections with *I* > 2σ(*I*)
*R*_int_ = 0.046

#### Refinement

*R*[*F* ^2^ > 2σ(*F* ^2^)] = 0.082
*wR*(*F* ^2^) = 0.229
*S* = 1.05
3434 reflections
181 parameters
1 restraint
H-atom parameters constrained
Δρ_max_ = 0.71 e Å^−3^
Δρ_min_ = −0.71 e Å^−3^

### 

Data collection: *CrystalClear* (Rigaku, 2005 ▶); cell refinement: *CrystalClear*; data reduction: *CrystalClear*; program(s) used to solve structure: *SHELXS97* (Sheldrick, 2008 ▶); program(s) used to refine structure: *SHELXL97* (Sheldrick, 2008 ▶); molecular graphics: *SHELXTL* (Sheldrick, 2008 ▶); software used to prepare material for publication: *SHELXTL*.

## Supplementary Material

Crystal structure: contains datablock(s) I, global. DOI: 10.1107/S160053681103892X/jh2327sup1.cif

Structure factors: contains datablock(s) I. DOI: 10.1107/S160053681103892X/jh2327Isup2.hkl

Supplementary material file. DOI: 10.1107/S160053681103892X/jh2327Isup3.cml

Additional supplementary materials:  crystallographic information; 3D view; checkCIF report

## Figures and Tables

**Table 1:** Hydrogen-bond geometry (Å, °)

*D*—H⋯*A*	*D*—H	H⋯*A*	*D*⋯*A*	*D*—H⋯*A*
N1—H1*A*⋯O1^i^	0.89	2.13	2.854 (4)	137
N1—H1*A*⋯S1^i^	0.89	2.94	3.794 (3)	161
N1—H1*B*⋯O1^ii^	0.89	1.89	2.777 (4)	175
N1—H1*C*⋯O2	0.89	2.01	2.773 (4)	143

Symmetry codes: (i) 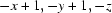; (ii) 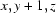.

**Fig. 1.**
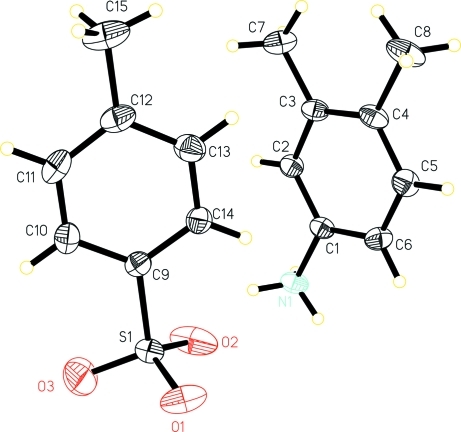
Perspective structure of the title compound, showing the atom-numbering scheme. Displacement ellipsoids are drawn at the 30% probability level.

**Fig. 2.**
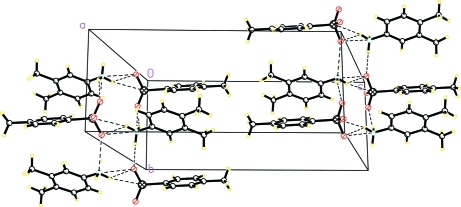
The crystal packing of the title compound viewed along the c axis showing the hydrogen bondings network.

## supplementary crystallographic information

### Comment

The title compound, was prepared as part of our ongoing studies of hydrogen-bonding interactions in the crystal structure of protonated amines. The importance of molecular salts as solid forms in pharmaceutical formulations is well known.For a given active ingredient, the isolation and selection of a salt with the appropriate physicochemical properties involves significant screening activity and has been discussed at some length in the literature (Tong & Whitesell *et al.* 1998; Shanker *et al.* 1994). Structures containing the dimethylanilinium cation have been already reported with tin chloride (Bouacida *et al.* 2008), sulfate (Singh *et al.* 2002), and dihydrogenphosphate. Here we report the synthesis and crystal structure of the title compound, 3,4-dimethylanilinium 4-methylbenzenesulfonate (Fig. 1).

The bond distances and bond angles in the title compound agree very well with the corresponding distances and angles reported for a closely related compound(Hemissi *et al.* 2001). In this structure, only one type of classical hydrogen bonds are observed, *viz*. cation–anion (Table 1). All three ammonium H atoms are involved in hydrogen bonds. These interactions result in the formation of cation-anion ribbons along c direction. Dipole-dipole and van der Waals interactions are effective in the molecular packing.

### Experimental

To a stirred solution of 3,4-dimethylbenzenamine (2.42 g, 0.02 mol) in 30 mL of methanol, 4-Toluene sulfonic acid (3.8 g, 0.02 mol) was added at the room temperature. The precipitate was filtered and washed with a small amount of ethanol 95%. Single crystals suitable for X-ray diffraction analysis were obtained from slow evaporation of a solution of the title compound in water at room temperature.

### Refinement

The H-atoms bonded to the C-atom were positioned geometrically and refined using a riding model, with C—H = 0.93–0.96 Å and *U*_iso_(H) = 1.2*U*_eq_(C). The H-atoms bonded to the N-atom were located from a difference map and were allowed to refine freely.

### Crystal data

**Table:**

C_8_H_12_N^+^C_7_H_7_O_3_S^−^	*F*(000) = 624
*M**_r_* = 293.37	*D*_x_ = 1.284 Mg m^−^^3^
Monoclinic, *P*2_1_/*n*	Mo *K*α radiation, λ = 0.71073 Å
Hall symbol: -P 2yn	Cell parameters from 3434 reflections
*a* = 12.373 (3) Å	θ = 2.6–27.4°
*b* = 7.3011 (15) Å	µ = 0.22 mm^−^^1^
*c* = 17.556 (4) Å	*T* = 293 K
β = 106.88 (3)°	Prism, colorless
*V* = 1517.7 (5) Å^3^	0.20 × 0.20 × 0.20 mm
*Z* = 4	

### Data collection

**Table:**

Rigaku Mercury2 diffractometer	3434 independent reflections
Radiation source: fine-focus sealed tube	2608 reflections with *I* > 2σ(*I*)
graphite	*R*_int_ = 0.046
Detector resolution: 13.6612 pixels mm^-1^	θ_max_ = 27.4°, θ_min_ = 3.0°
CCD_Profile_fitting scans	*h* = −15→15
Absorption correction: multi-scan (*CrystalClear*; Rigaku, 2005)	*k* = −9→9
*T*_min_ = 0.825, *T*_max_ = 1.000	*l* = −22→22
14838 measured reflections	

### Refinement

**Table:**

Refinement on *F*^2^	Primary atom site location: structure-invariant direct methods
Least-squares matrix: full	Secondary atom site location: difference Fourier map
*R*[*F*^2^ > 2σ(*F*^2^)] = 0.082	Hydrogen site location: inferred from neighbouring sites
*wR*(*F*^2^) = 0.229	H-atom parameters constrained
*S* = 1.05	*w* = 1/[σ^2^(*F*_o_^2^) + (0.123*P*)^2^ + 1.5301*P*] where *P* = (*F*_o_^2^ + 2*F*_c_^2^)/3
3434 reflections	(Δ/σ)_max_ < 0.001
181 parameters	Δρ_max_ = 0.71 e Å^−^^3^
1 restraint	Δρ_min_ = −0.71 e Å^−^^3^

### Special details

**Table:**

Geometry. All e.s.d.'s (except the e.s.d. in the dihedral angle between two l.s. planes) are estimated using the full covariance matrix. The cell e.s.d.'s are taken into account individually in the estimation of e.s.d.'s in distances, angles and torsion angles; correlations between e.s.d.'s in cell parameters are only used when they are defined by crystal symmetry. An approximate (isotropic) treatment of cell e.s.d.'s is used for estimating e.s.d.'s involving l.s. planes.
Refinement. Refinement of *F*^2^ against ALL reflections. The weighted *R*-factor *wR* and goodness of fit *S* are based on *F*^2^, conventional *R*-factors *R* are based on *F*, with *F* set to zero for negative *F*^2^. The threshold expression of *F*^2^ > σ(*F*^2^) is used only for calculating *R*-factors(gt) *etc*. and is not relevant to the choice of reflections for refinement. *R*-factors based on *F*^2^ are statistically about twice as large as those based on *F*, and *R*- factors based on ALL data will be even larger.

### Fractional atomic coordinates and isotropic or equivalent isotropic displacement parameters (Å^2^)

**Table:**

	*x*	*y*	*z*	*U*_iso_*/*U*_eq_	
S1	0.32958 (7)	0.27384 (11)	0.06233 (5)	0.0414 (3)	
N1	0.4796 (2)	0.7784 (4)	0.07083 (15)	0.0429 (6)	
H1A	0.5143	0.7406	0.0358	0.064*	
H1B	0.4597	0.8952	0.0615	0.064*	
H1C	0.4182	0.7105	0.0661	0.064*	
C3	0.5882 (3)	0.7845 (4)	0.29494 (18)	0.0386 (7)	
C4	0.6969 (3)	0.7113 (4)	0.30705 (18)	0.0413 (7)	
C1	0.5569 (3)	0.7596 (4)	0.15244 (17)	0.0346 (6)	
C9	0.3539 (3)	0.2660 (4)	0.16787 (19)	0.0373 (7)	
C10	0.2706 (3)	0.1959 (5)	0.1992 (2)	0.0473 (8)	
H10A	0.2031	0.1522	0.1654	0.057*	
C2	0.5186 (3)	0.8113 (4)	0.21675 (17)	0.0368 (7)	
H2A	0.4474	0.8632	0.2079	0.044*	
C6	0.6634 (3)	0.6868 (4)	0.16339 (19)	0.0424 (7)	
H6A	0.6880	0.6540	0.1200	0.051*	
C14	0.4558 (3)	0.3289 (4)	0.21888 (19)	0.0430 (7)	
H14A	0.5119	0.3735	0.1983	0.052*	
C12	0.3897 (4)	0.2572 (5)	0.3338 (2)	0.0511 (9)	
O1	0.4053 (3)	0.1381 (4)	0.04418 (16)	0.0717 (9)	
C5	0.7330 (3)	0.6636 (5)	0.2409 (2)	0.0447 (8)	
H5A	0.8051	0.6155	0.2490	0.054*	
C13	0.4728 (3)	0.3244 (5)	0.3013 (2)	0.0513 (9)	
H13A	0.5407	0.3668	0.3351	0.062*	
C7	0.5443 (4)	0.8386 (6)	0.3646 (2)	0.0596 (10)	
H7A	0.6009	0.8124	0.4138	0.089*	
H7B	0.4771	0.7703	0.3621	0.089*	
H7C	0.5275	0.9672	0.3618	0.089*	
C11	0.2890 (3)	0.1918 (5)	0.2808 (2)	0.0544 (9)	
H11A	0.2332	0.1446	0.3011	0.065*	
O2	0.3571 (4)	0.4547 (4)	0.04248 (16)	0.0895 (12)	
C15	0.4082 (5)	0.2539 (7)	0.4234 (3)	0.0796 (15)	
H15A	0.4811	0.3042	0.4499	0.119*	
H15B	0.4044	0.1299	0.4406	0.119*	
H15C	0.3508	0.3255	0.4362	0.119*	
C8	0.7761 (3)	0.6797 (6)	0.3905 (2)	0.0631 (11)	
H8A	0.7398	0.7184	0.4293	0.095*	
H8B	0.8441	0.7491	0.3972	0.095*	
H8C	0.7943	0.5519	0.3977	0.095*	
O3	0.2122 (3)	0.2272 (6)	0.0250 (2)	0.1043 (14)	

### Atomic displacement parameters (Å^2^)

**Table:**

	*U*^11^	*U*^22^	*U*^33^	*U*^12^	*U*^13^	*U*^23^
S1	0.0502 (5)	0.0355 (4)	0.0360 (4)	0.0014 (3)	0.0084 (3)	0.0001 (3)
N1	0.0532 (16)	0.0414 (14)	0.0281 (12)	0.0017 (12)	0.0025 (11)	0.0021 (10)
C3	0.0493 (17)	0.0337 (15)	0.0296 (14)	0.0016 (13)	0.0064 (13)	0.0014 (11)
C4	0.0497 (18)	0.0320 (15)	0.0338 (15)	0.0037 (13)	−0.0010 (13)	0.0018 (12)
C1	0.0418 (16)	0.0298 (14)	0.0282 (13)	−0.0003 (11)	0.0039 (12)	0.0038 (10)
C9	0.0439 (17)	0.0309 (14)	0.0396 (15)	0.0010 (12)	0.0160 (13)	0.0003 (12)
C10	0.0421 (17)	0.0441 (18)	0.059 (2)	−0.0026 (14)	0.0198 (16)	0.0010 (15)
C2	0.0377 (15)	0.0361 (15)	0.0344 (15)	0.0023 (12)	0.0070 (12)	0.0029 (12)
C6	0.0503 (18)	0.0408 (17)	0.0369 (16)	0.0075 (14)	0.0138 (14)	−0.0016 (13)
C14	0.0473 (17)	0.0422 (17)	0.0421 (17)	−0.0079 (14)	0.0170 (14)	0.0006 (13)
C12	0.075 (3)	0.0411 (18)	0.0438 (18)	0.0071 (17)	0.0276 (18)	0.0044 (14)
O1	0.111 (2)	0.0637 (18)	0.0456 (14)	0.0344 (17)	0.0310 (16)	0.0082 (13)
C5	0.0414 (17)	0.0413 (17)	0.0469 (18)	0.0109 (13)	0.0059 (14)	0.0006 (14)
C13	0.059 (2)	0.051 (2)	0.0405 (17)	−0.0064 (16)	0.0094 (16)	0.0003 (15)
C7	0.077 (3)	0.066 (2)	0.0380 (18)	0.007 (2)	0.0204 (18)	0.0005 (17)
C11	0.058 (2)	0.053 (2)	0.063 (2)	0.0037 (17)	0.0359 (19)	0.0102 (17)
O2	0.171 (4)	0.0434 (16)	0.0445 (15)	−0.0182 (19)	0.0171 (19)	0.0062 (12)
C15	0.121 (4)	0.080 (3)	0.045 (2)	0.016 (3)	0.036 (3)	0.008 (2)
C8	0.073 (3)	0.057 (2)	0.0400 (18)	0.0121 (19)	−0.0142 (18)	−0.0014 (16)
O3	0.057 (2)	0.180 (4)	0.062 (2)	−0.021 (2)	−0.0047 (16)	−0.005 (2)

### Geometric parameters (Å, °)

**Table:**

S1—O2	1.432 (3)	C6—C5	1.394 (5)
S1—O3	1.449 (3)	C6—H6A	0.9300
S1—O1	1.461 (3)	C14—C13	1.400 (5)
S1—C9	1.790 (3)	C14—H14A	0.9300
N1—C1	1.480 (4)	C12—C13	1.402 (5)
N1—H1A	0.8900	C12—C11	1.405 (6)
N1—H1B	0.8900	C12—C15	1.523 (5)
N1—H1C	0.8900	C5—H5A	0.9300
C3—C4	1.405 (5)	C13—H13A	0.9300
C3—C2	1.406 (4)	C7—H7A	0.9600
C3—C7	1.527 (5)	C7—H7B	0.9600
C4—C5	1.404 (5)	C7—H7C	0.9600
C4—C8	1.526 (4)	C11—H11A	0.9300
C1—C6	1.381 (4)	C15—H15A	0.9600
C1—C2	1.397 (4)	C15—H15B	0.9600
C9—C14	1.396 (5)	C15—H15C	0.9600
C9—C10	1.399 (4)	C8—H8A	0.9600
C10—C11	1.384 (5)	C8—H8B	0.9600
C10—H10A	0.9300	C8—H8C	0.9600
C2—H2A	0.9300		
			
O2—S1—O3	112.6 (2)	C9—C14—C13	119.5 (3)
O2—S1—O1	111.1 (2)	C9—C14—H14A	120.3
O3—S1—O1	111.3 (2)	C13—C14—H14A	120.3
O2—S1—C9	107.53 (15)	C13—C12—C11	117.6 (3)
O3—S1—C9	107.81 (19)	C13—C12—C15	121.2 (4)
O1—S1—C9	106.16 (15)	C11—C12—C15	121.2 (4)
C1—N1—H1A	109.5	C6—C5—C4	121.5 (3)
C1—N1—H1B	109.5	C6—C5—H5A	119.3
H1A—N1—H1B	109.5	C4—C5—H5A	119.3
C1—N1—H1C	109.5	C14—C13—C12	121.4 (3)
H1A—N1—H1C	109.5	C14—C13—H13A	119.3
H1B—N1—H1C	109.5	C12—C13—H13A	119.3
C4—C3—C2	119.2 (3)	C3—C7—H7A	109.5
C4—C3—C7	121.6 (3)	C3—C7—H7B	109.5
C2—C3—C7	119.1 (3)	H7A—C7—H7B	109.5
C5—C4—C3	119.3 (3)	C3—C7—H7C	109.5
C5—C4—C8	119.1 (3)	H7A—C7—H7C	109.5
C3—C4—C8	121.5 (3)	H7B—C7—H7C	109.5
C6—C1—C2	121.7 (3)	C10—C11—C12	121.7 (3)
C6—C1—N1	119.5 (3)	C10—C11—H11A	119.1
C2—C1—N1	118.8 (3)	C12—C11—H11A	119.1
C14—C9—C10	120.0 (3)	C12—C15—H15A	109.5
C14—C9—S1	120.2 (2)	C12—C15—H15B	109.5
C10—C9—S1	119.8 (3)	H15A—C15—H15B	109.5
C11—C10—C9	119.8 (3)	C12—C15—H15C	109.5
C11—C10—H10A	120.1	H15A—C15—H15C	109.5
C9—C10—H10A	120.1	H15B—C15—H15C	109.5
C1—C2—C3	119.7 (3)	C4—C8—H8A	109.5
C1—C2—H2A	120.1	C4—C8—H8B	109.5
C3—C2—H2A	120.1	H8A—C8—H8B	109.5
C1—C6—C5	118.5 (3)	C4—C8—H8C	109.5
C1—C6—H6A	120.8	H8A—C8—H8C	109.5
C5—C6—H6A	120.8	H8B—C8—H8C	109.5

### Hydrogen-bond geometry (Å, °)

**Table:**

*D*—H···*A*	*D*—H	H···*A*	*D*···*A*	*D*—H···*A*
N1—H1A···O1^i^	0.89	2.13	2.854 (4)	137.
N1—H1A···S1^i^	0.89	2.94	3.794 (3)	161.
N1—H1B···O1^ii^	0.89	1.89	2.777 (4)	175.
N1—H1C···O2	0.89	2.01	2.773 (4)	143.

Symmetry codes: (i) −*x*+1, −*y*+1, −*z*; (ii) *x*, *y*+1, *z*.
